# A conjecture of Warnaar-Zudilin from deformations of lie superalgebras

**DOI:** 10.1007/s40687-026-00613-2

**Published:** 2026-04-02

**Authors:** Thomas Creutzig, Niklas Garner

**Affiliations:** 1 Department of Mathematics, FAU Erlangen, 91058 Erlangen, Germany; 2 Mathematical Institute, University of Oxford, Oxford, OX2 6GG UK

**Keywords:** Primary 05A19, 17B65, 17B69

## Abstract

We prove a collection of *q*-series identities conjectured by Warnaar and Zudilin and appearing in recent work with H. Kim in the context of superconformal field theory. Our proof utilizes a deformation of the simple affine vertex operator superalgebra $L_k(\mathfrak {osp}_{1|2n})$ into the principal subsuperspace of $L_k({\mathfrak {s}}{\mathfrak {l}}_{1|2n+1})$ in a manner analogous to earlier work of Feigin-Stoyanovsky. This result fills a gap left by Stoyanovsky, showing that for all positive integers *N*, *k* the character of the principal subspace of type $A_N$ at level *k* can be identified with the (super)character of a simple affine vertex operator (super)algebra at the same level.

## Introduction

Vertex operator algebras (VOAs) appear in a wide variety of physical problems and are a rich source of mathematics that serve as a fertile bridge between algebra and number theory. In this paper, we capitalize on insight gleaned from a recent physical analysis [6] with H. Kim to establish a family of *q*-series identities of interest to number theorists.

### Theorem 1.1

(Main result, Corollary 4.8) For every positive integer *n*, *k* the following is an equality of *q*-series:

1.1 $$\begin{aligned} \frac{1}{(q)_\infty ^{n(2n-1)}}\sum _{u \in {{\textbf{Z}}}^n}(-1)^{|u|} \xi (u) q^{(k+n+\scriptstyle {\frac{1}{2}})|\!|u|\!|^2+ \rho \cdot u} = \sum _{m \in {\mathbb {N}}^{k(2n-1)}} \frac{q^{\scriptstyle {\frac{1}{2}}\sum \limits _{i,j=1}^{k}\sum \limits _{a,b=1}^{2n-1} T_{ij} A_{ab} m_{ia}m_{jb}}}{\prod \limits _{i=1}^k \prod \limits _{a=1}^{2n-1} (q)_{m_{ia}}} \end{aligned}$$

In this theorem, the matrices $A_{ab}$ and $T_{ij}$ are simply the Cartan matrix of $A_{2n-1}$ and the inverse of the Cartan matrix of the rank *k* tadpole graph

$$ A_{ab} = {\left\{ \begin{array}{ll} 2 &  a = b\\ -1 &  a = b \pm 1\\ 0 &  \text {else} \end{array}\right. } \qquad \text {and} \qquad T_{ij} = \min (i,j)\,, $$

|*u*| denotes the sum of the components of *u*, $$|\!|u|\!|^2 = u \cdot u$$ the squared norm of *u*, $$\rho $$ the vector with components $$\rho _m = m - \frac{1}{2}$$, and finally

$$ \xi (u) = \prod _{1 \le l < m \le n} \frac{v_m^2 - v_l^2}{\rho _m^2 - \rho _l^2} \qquad v_m = \rho _m + (2(n+k)+1)u_m\,. $$

This collection of *q*-series identities was proved for $k = 1$ and conjectured to hold more generally by Warnaar and Zudilin [23, Conjecture 1.1, Theorem 1.2] and were viewed by these authors as an extension of the Rogers-Ramanujan [2, 17] (for $n = k = 1$) and Andrews-Gordon [1, 11] (for $n = 1$) identities and a specialization of the $A^{(2)}_{2n}$ Macdonald identities [16] (for $k = 0$, where the right-hand side reduces to 1).^1^

We instead view the *q*-series appearing on the two sides of the Eq. (1.1) as supercharacters of VOAs. The left-hand side of Eq. (1.1) is the supercharacter of the simple affine VOA $L_k(\mathfrak {osp}_{1|2n})$; see Appendix A for more details. The right-hand side of Eq. (1.1) is the character of a different VOA: as conjectured (and proven for ${\mathfrak {s}}{\mathfrak {l}}_2$) by Feigin-Stoyanovsky [21] and proven more generally by Georgiev [10], it is the character of the principal subspace of $L_k({\mathfrak {s}}{\mathfrak {l}}_{2n})$; we build on these results to show that it is also the supercharacter of the principal subspace of $L_k({\mathfrak {s}}{\mathfrak {l}}_{1|2n+1})$. An analogous family of *q*-series identities appeared in work of Stoyanovsky [22] (where the left-hand side is replaced by the character of the simple affine VOA $L_k({\mathfrak {s}}{\mathfrak {p}}_{2n})$ and the right-hand side is replaced by the character of the principal subspace of $L_k({\mathfrak {s}}{\mathfrak {l}}_{2n+1})$) by showing that ${\mathfrak {s}}{\mathfrak {p}}_{2n}$ can be deformed into a nilpotent subalgebra of ${\mathfrak {s}}{\mathfrak {l}}_{2n+1}$, that this deformation can be extended to the affine setting, and that its character does not change under this deformation. We establish Eq. (1.1) by similarly deforming $\mathfrak {osp}_{1|2n}$ into a nilpotent subalgebra of ${\mathfrak {s}}{\mathfrak {l}}_{1|2n+1}$. The equality of the *q*-series in Eq. (1.1) is thus an immediate consequence of the following theorem:

### Theorem 1.2

(Theorem 4.6) The supercharacter of $L_k(\mathfrak {osp}_{1|2n})$ is equal to both the supercharacter of the principal subspace of $L_k({\mathfrak {s}}{\mathfrak {l}}_{1|2n+1})$ and to the character of the principal subspace of $L_k({\mathfrak {s}}{\mathfrak {l}}_{2n})$.

Our result fills a gap left by Stoyanovsky, i.e. that the vacuum character of the principal subspace of $L_k({\mathfrak {s}}{\mathfrak {l}}_{N})$ can be identified with the vacuum character of a simple affine VOA $L_k(\mathfrak {g}_N)$ for all integers $N \ge 2$ and $k \ge 1$, where $\mathfrak {g}_{2n+1} \simeq {\mathfrak {s}}{\mathfrak {p}}_{2n}$ and $\mathfrak {g}_{2n} \simeq \mathfrak {osp}_{1|2n}$. We also note that an immediate corollary of this result establishes part of a conjecture of Bringmann, Calinescu, Folsom, and Kimport concerning the modularity of the right-hand side of Eq. (1.1). Explicitly, together with their Theorem 1.2 this result proves Case i. of Conjecture 4.1 of [3] for *N* even and $\ell = k$, cf. Remark 7 of loc. cit.

## Deformations of Lie superalgebras

In this section we study certain deformations of the simple Lie algebras ${\mathfrak {s}}{\mathfrak {p}}_{2n}$, ${\mathfrak {s}}{\mathfrak {o}}_m$, and $\mathfrak {osp}_{m|2n}$ into principal nilpotent subalgebras of ${\mathfrak {s}}{\mathfrak {l}}_{2n+1}$, ${\mathfrak {s}}{\mathfrak {l}}_{m}$, and ${\mathfrak {s}}{\mathfrak {l}}_{m|2n+1}$, respectively. The first two examples are meant to elaborate on [22], and the last example is a simple generalization of the construction.

### ${\mathfrak {s}}{\mathfrak {p}}_{2n}$

We start with the main example of [22]. Consider the (odd) vector space ${{\textbf{C}}}^{0|2n+1} = \Pi {{\textbf{C}}}^{2n+1}$ and choose a basis $\theta _a$ for $a = 0, \dots , 2n$. We introduce a (degenerate) supersymmetric bilinear form on ${{\textbf{C}}}^{0|2n+1}[x]:= {{\textbf{C}}}^{0|2n+1} \otimes _{{\textbf{C}}}{{\textbf{C}}}[x]$ given by the (super)symmetric quadratic tensor

$$ B = \sum _{a=0}^{2n-1} x^a \theta _a \theta _{a+1}\,. $$

We then consider the family of Lie algebras $\mathfrak {g}$ defined as the subalgebra of ${\mathfrak {s}}{\mathfrak {l}}_{0|2n+1}[x] \simeq {\mathfrak {s}}{\mathfrak {l}}_{2n+1}[x]$ that preserves $\theta _0$ and *B*; set $\mathfrak {g}_\epsilon := \mathfrak {g}\otimes _{{{\textbf{C}}}[x]} {{\textbf{C}}}[x]/(x - \epsilon )$. We aim to show that for $\epsilon \ne 0$ this Lie algebra can be identified with ${\mathfrak {s}}{\mathfrak {p}}_{2n}$ and that for $\epsilon = 0$ the subalgebra tends to the nilpotent subalgebra of ${\mathfrak {s}}{\mathfrak {l}}_{2n+1}$.

#### Example 2.1

For $n = 1$ the algebra $\mathfrak {g}$ is spanned (as a ${{\textbf{C}}}[x]$-module) by

$$ h_x = \begin{pmatrix} 0 &  0 &  1\\ 0 &  x &  0\\ 0 &  0 &  -x \end{pmatrix} \qquad e_x = \begin{pmatrix} 0 &  0 &  0\\ 0 &  0 &  -1\\ 0 &  0 &  0 \end{pmatrix} \qquad f_x = \begin{pmatrix} 0 &  1 &  0\\ 0 &  0 &  0\\ 0 &  -x &  0 \end{pmatrix} $$

which satisfy the following commutation relations:

$$ [h_x, e_x] = 2 x e_x \qquad [h_x, f_x] = - 2 x f_x \qquad [e_x, f_x] = h_x $$

It is evident from these commutators that for non-zero $\epsilon$ the algebra $\mathfrak {g}_\epsilon$ is precisely ${\mathfrak {s}}{\mathfrak {p}}_2$. Moreover, $\mathfrak {g}_0$ is the strictly upper-triangular subalgebra.

#### Lemma 2.2

$\mathfrak {g}_0$ is isomorphic to the strictly upper triangular subalgebra of ${\mathfrak {s}}{\mathfrak {l}}_{2n+1}$.

#### Proof

We make explicit the constraints imposed by invariance of $\theta _0$ and *B* under $X \in {\mathfrak {s}}{\mathfrak {l}}_{2n+1}[x]$. Let  be the matrix elements of *X* in the basis $\theta _a$. Preserving $\theta _0$ implies  and preserving $B_x$ implies2.1for all $a, b = 0, \dots 2n$, where we set  and ; it suffices to consider $a < b$ due to the anti-symmetry of this constraint. We will deduce the lemma by showing that these constraints imply  is unconstrained for $a < b$ and belongs to $x {{\textbf{C}}}[x]$ otherwise.

We proceed by induction. Consider first the case $a = 0$, where Eq. (2.1) impliesfor all $1 \le b \le 2n$. This is manifestly proportional to *x* for $b > 1$; this also holds for $b = 1$ because . In particular,  is constrained to be a (linear) polynomial of the strictly upper-triangular matrix elements for all *b* and is proportional to $x^{b-1}$ for $b > 1$ and proportional to *x* for $b = 1$.

Now suppose for all $a \le a_0$ and $b \ge a$ we have shown that the matrix element  is constrained to be a (linear) polynomial in the strictly upper-triangular matrix elements that is proportional to $x^{b-a}$ for all $b > a$ and proportional to *x* for $b = a$. Rearranging Eq. (2.1) for $a = a_0$ givesThe inductive hypothesis implies that the right-hand side is necessarily a (linear) polynomial in the strictly upper-triangular matrix elements. Moreover, the first term is proportional to $x^{b-a_0}$ and the second term is proportional to $x^{b-a_0-1}$ for all $b > a_0+1$ and proportional to *x* for $b = a_0+1$, as desired. $\square$

#### Remark 2.3

We make the solution to the above constraints explicit for $n = 2$. A general element *X* takes the form

#### Lemma 2.4

$\mathfrak {g}_\epsilon$ can be identified with ${\mathfrak {s}}{\mathfrak {p}}_{2n}$ when $\epsilon \ne 0$.

#### Proof

We consider the following elements of ${{\textbf{C}}}^{0|2n+1}\otimes _{{\textbf{C}}}[x]$:

$$ \theta = \theta _0 \qquad \phi _{2i-1} = x^{i-1} \theta _{2i-1} \qquad \phi _{2i} = x^{i-1}(x \theta _{2i} - \theta _{2i-2}) $$

where $i = 1, \dots , n$; these elements are conjugate to the basis $\theta _a$ if we localize *x*, i.e. they are conjugate bases of ${{\textbf{C}}}^{0|2n+1}[x^{\pm 1}]$ as a ${{\textbf{C}}}[x^{\pm 1}]$-module. In this basis *B* takes the form

$$ B = \sum _{i=1}^{n} \phi _{2i-1} \phi _{2i} $$

which is non-degenerate when restricted to the subspace spanned by the $\phi$’s. In particular, we conclude that the subalgebra of ${\mathfrak {s}}{\mathfrak {l}}_{2n+1}[x^{\pm 1}]$ preserving $\theta = \theta _0$ and *B* is precisely the ${\mathfrak {s}}{\mathfrak {p}}_{2n}[x^{\pm 1}]$ rotating the $\phi$’s. $\square$

#### Remark 2.5

The original basis $\theta _a$ is not a weight basis for the Cartan subalgebra of ${\mathfrak {s}}{\mathfrak {p}}_{2n}$ rotating the pairs $\phi _{2i-1}, \phi _{2i}$ with opposite weights and preserving $\theta$. Both are eigenbases for the linear transformation that scales $\theta _a$ for *a* odd (resp. even) with weight 1 (resp. $-1$); this transformation preserves *B*, but fails to preserve $\theta _0$ (only preserving its span) and does not belong to ${\mathfrak {s}}{\mathfrak {l}}_{2n+1}$ as it has trace 1. Nonetheless, it induces a $2{{\textbf{Z}}}$-grading on the family $\mathfrak {g}_\epsilon$. For $n = 1$ and $\epsilon \ne 0$ it can be identified with the $h_x$ weight grading.

### ${\mathfrak {s}}{\mathfrak {o}}_{m}$

We now describe the example mentioned in Remark 2 of [22], suggested to the author by Panov. We consider the (even) vector space ${{\textbf{C}}}^{m|0} = {{\textbf{C}}}^m$ and choose a basis $z_r$, $r = 1, \dots , m$. We then consider the symmetric quadratic tensor

$$ C = \tfrac{1}{2}\sum _{r=1}^{m} x^{2(r-1)} z_{r}^2\,. $$

We define the Lie algebra $\mathfrak {f}$ as the subalgebra of ${\mathfrak {s}}{\mathfrak {l}}_{m|0}[x] \simeq {\mathfrak {s}}{\mathfrak {l}}_m[x]$ preserving *C* and denote $\mathfrak {f}_\epsilon := \mathfrak {f}\otimes _{{{\textbf{C}}}[x]} {{\textbf{C}}}[x]/(x - \epsilon )$. The analog of Eq. (2.1) for  takes the form2.2which implies  for all $s \ge r$. We can similarly consider the conjugate basis $w_r = x^{r-1} z_r$, where the quadratic tensor takes the form

$$ C = \tfrac{1}{2}\sum _{r=1}^{m} w_{r}^2\,. $$

#### Lemma 2.6

$\mathfrak {f}_\epsilon$ is isomorphic to the strictly upper-triangular subalgebra of ${\mathfrak {s}}{\mathfrak {l}}_{m}$ for $\epsilon = 0$ and isomorphic to ${\mathfrak {s}}{\mathfrak {o}}_{m}$ for $\epsilon \ne 0$.

### $\mathfrak {osp}_{m|2n}$

We now combine the above examples and consider the (super) vector space ${{\textbf{C}}}^{m|2n+1} = {{\textbf{C}}}^m \oplus \Pi {{\textbf{C}}}^{2n+1}$ with (homogeneous) basis $z_r, \theta _a$. We then consider the (super)symmetric quadratic tensor

$$ D = \tfrac{1}{2}\sum _{r=1}^m x^{2(r-1)} z^2_{r} + x^{2m} \sum _{a=0}^{2n-1} x^{2a} \theta _a \theta _{a+1} $$

We define the Lie superalgebra $\mathfrak {k}$ as the subalgebra of ${\mathfrak {s}}{\mathfrak {l}}_{m|2n+1}[x]$ preserving $\theta _0$ and *D* and define $\mathfrak {k}_\epsilon$ as before.

#### Example 2.7

For the case $m = n = 1$ we find that $\mathfrak {k}$ has even generators

$$ h_x = \left( \begin{array}{c | c c c} 0 &  0 &  0 &  0\\ \hline 0 &  0 &  0 &  1\\ 0 &  0 &  x^2 &  0\\ 0 &  0 &  0 &  -x^2\\ \end{array}\right) \qquad e_x = \left( \begin{array}{c | c c c} 0 &  0 &  0 &  0\\ \hline 0 &  0 &  0 &  0\\ 0 &  0 &  0 &  -1\\ 0 &  0 &  0 &  0\\ \end{array}\right) \qquad f_x = \left( \begin{array}{c | c c c} 0 &  0 &  0 &  0\\ \hline 0 &  0 &  1 &  0\\ 0 &  0 &  0 &  0\\ 0 &  0 &  -x^2 &  0\\ \end{array}\right) $$

and odd generators

$$ \psi _{1,x} = \left( \begin{array}{c | c c c} 0 &  0 &  0 &  1\\ \hline 0 &  0 &  0 &  0\\ x^4 &  0 &  0 &  0\\ 0 &  0 &  0 &  0\\ \end{array}\right) \qquad \psi _{2,x} = \left( \begin{array}{c | c c c} 0 &  0 &  1 &  0\\ \hline x^2 &  0 &  0 &  0\\ 0 &  0 &  0 &  0\\ -x^4 &  0 &  0 &  0\\ \end{array}\right) $$

#### Lemma 2.8

The subalgebra $\mathfrak {k}_\epsilon$ is isomorphic to the strictly upper-triangular subalgebra of ${\mathfrak {s}}{\mathfrak {l}}_{m|2n+1}$ preserving $\theta _0$ for $\epsilon = 0$ and is isomorphic to $\mathfrak {osp}_{m|2n}$ when $\epsilon \ne 0$.

#### Remark 2.9

As is evident in the above example, the subalgebra $\mathfrak {k}_\epsilon$ does not deform to the entire strictly upper-triangular subalgebra of ${\mathfrak {s}}{\mathfrak {l}}_{m|2n+1}$ unless $m = 0$. Indeed, the odd subspace of the latter has dimension $m(2n+1)$ whereas the odd subspace of $\mathfrak {osp}_{m|2n}$ is dimension 2*mn*.

#### Proof

The second assertion follows from the changes of basis introduced in Sections 2.1 and 2.2.

To establish the first assertion, we note that the even subalgebra of $\mathfrak {k}$ is simply $\mathfrak {f}\oplus \mathfrak {g}$ — the additional even generator on top of ${\mathfrak {s}}{\mathfrak {l}}_m[x] \oplus {\mathfrak {s}}{\mathfrak {l}}_{2n+1}[x]$ does not preserve $\theta _0$. We see that the even subalgebra of $\mathfrak {k}_0$ is simply the nilpotent subalgebra of ${\mathfrak {s}}{\mathfrak {l}}_m \oplus {\mathfrak {s}}{\mathfrak {l}}_{2n+1}$. To check the odd subspace of $\mathfrak {k}_0$, we consider (odd) linear map sending  and , with ; we must verify that  is proportional to *x*. Preserving *D* requiresfor $a = 0, \dots , 2n$ and $r = 1, \dots , m$, where we set  and . In particular, we must that have  is proportional to *x* for all *a*, *r*. $\square$

## Duflo-Serganova reduction of deformations

The following is a special case of cohomological reduction [5] as well as the Duflo-Serganova functor [12, 20].

### ${\mathfrak {g}}{\mathfrak {l}}_{1|1}$ preliminaries

We fix a basis $N, E, \psi ^\pm$ of ${\mathfrak {g}}{\mathfrak {l}}_{1|1}$ with even generators *N*, *E*, odd generators $\psi ^\pm$, and commutation relations

$$ [N, \psi ^\pm ] = \pm \psi ^\pm , \qquad [\psi ^+, \psi ^-] = E $$

and *E* the central element of ${\mathfrak {g}}{\mathfrak {l}}_{1|1}$. We call a ${\mathfrak {g}}{\mathfrak {l}}_{1|1}$-module a weight module if its Cartan subalgebra acts semisimply. As it is central, *E* acts on an indecomposable module by multiplication by a scalar and so the category of weight modules admits a block decompositon

$$ {{\mathcal {C}}}= \bigoplus _{e \in {{\textbf{C}}}} {{\mathcal {C}}}_e $$

with ${{\mathcal {C}}}_e$ the block on which *E* acts by the scalar *e*. Let ${{\mathcal {D}}}_0$ be the category whose objects are direct sums of trivial representations of ${\mathfrak {g}}{\mathfrak {l}}_{1|1}$ and set

$$ {{\mathcal {D}}}:= {{\mathcal {D}}}_0 \oplus \bigoplus _{e \in {{\textbf{R}}}_{>0}} {{\mathcal {C}}}_e. $$

This category is semisimple and closed under tensor products. We complete this category to allow for countable direct sums of objects and denote this completion by the symbol $\overline{{{\mathcal {D}}}}$.

For *M* an object of ${{\mathcal {D}}}$, let *H*(*M*) denote the cohomology of *M* with respect to $\psi ^+$.

#### Lemma 3.1

Let *M* be an object in $\overline{{{\mathcal {D}}}}$ and let

$$ M = \bigoplus _{e \in {{\textbf{R}}}_{\ge 0}} M_e $$

be its decomposition into *E*-eigenspaces. Then $H(M) = M_0$, and if *M* in ${{\mathcal {D}}}$, then also $\text {sdim}(M) = \text {sdim}(M_0)$.

#### Proof

Let $e\ne 0$, then a simple object $M_{n, e}$ in ${{\mathcal {C}}}_e$ is two dimensional. It is generated by a highest-weight vector *v*, such that $Nv = nv$, $Ev=ev$ and $\psi ^+v =0$. The module $M_{n,e}$ is spanned by $v, \psi ^-v$ and hence has super dimension zero. Moreover, $\psi ^+\psi ^-v = [\psi ^+, \psi ^-]v = Ev = ev$ and so $H(M_{n, e}) = 0$.

For a general object *M* of $\overline{{{\mathcal {D}}}}$, because $\overline{{{\mathcal {D}}}}$ is semisimple it follows that the submodule $M_e$ for $e\ne 0$ is a countable direct sum of $M_{n,e}$ (typically with different values of *n* appearing) and hence $H(M_e) = 0$ and $\text {sdim}(M_e) = 0$. As the action of $\psi ^+$ is trivial on the submodule $M_0$ we conclude

$$ H(M) = \bigoplus _e H(M_e) = H(M_0) = M_0 $$

and additionally if *M* in ${{\mathcal {D}}}$, $\text {sdim}(M) = \text {sdim}(M_0)$. $\square$

### Formalization of the deformation problem

In this section we formalize our problem. Let $\mathfrak {g}$ be a Lie superalgebra with non-degenerate bilinear form $\kappa$ and let $\mathfrak {a}$ be a subalgebra of $\mathfrak {g}\otimes _{{\textbf{C}}}{{\textbf{C}}}[x]$. Let $\mathfrak {a}_\epsilon = \mathfrak {a}\otimes _{{{\textbf{C}}}[x]} {{\textbf{C}}}[x]/(x-\epsilon )$ be a 1-parameter family of subalgebras; set $\mathfrak {p}:= \mathfrak {a}_0$. We denote by $\widehat{\mathfrak {g}}$ the central extension

$$ 0 \rightarrow {{\textbf{C}}}K \rightarrow \widehat{\mathfrak {g}} \rightarrow \mathfrak {g}\otimes {{\textbf{C}}}[t^{\pm 1}] \rightarrow 0 $$

induced by the bilinear form $\kappa$ and similarly denote $\widehat{\mathfrak {p}} = \mathfrak {p}\otimes {{\textbf{C}}}[t^{\pm 1}]$. Let $V^k(\mathfrak {g})$ be the universal affine VOA of $\mathfrak {g}$ at level $k\in {{\textbf{C}}}$ associated to $\kappa$ and let $V^k(\mathfrak {a}_\epsilon )$, $V^k(\mathfrak {p})$ be the affine subVOAs associated to $\mathfrak {a}_\epsilon$, $\mathfrak {p}$. For *I* an ideal in $V^k(\mathfrak {g})$, we set $I_\epsilon = I \cap V^k(\mathfrak {a}_\epsilon )$ and $I_\mathfrak {p}:= I_0$. Denote the quotients by the corresponding ideals by $L_k(\mathfrak {g}), L_k(\mathfrak {a}_\epsilon ), L_k(\mathfrak {p})$. There is thus a short exact sequence

$$ 0 \rightarrow I_\mathfrak {p}\rightarrow V^k(\mathfrak {p}) \rightarrow L_k(\mathfrak {p}) \rightarrow 0. $$

Assume that there is an embedding $\iota : {\mathfrak {g}}{\mathfrak {l}}_{1|1} \hookrightarrow \mathfrak {g}$ such that ${\mathfrak {g}}{\mathfrak {l}}_{1|1}$ acts on $\mathfrak {p}$

$$ [\iota (x), y] \in \mathfrak {p}\qquad \forall \ x \in {\mathfrak {g}}{\mathfrak {l}}_{1|1}, \ y \in \mathfrak {p}. $$

This action extends in the obvious way to $\widehat{\mathfrak {p}}$.

#### Lemma 3.2

With the above set-up, if $\mathfrak {p}$ is an object in ${{\mathcal {D}}}$, then

$$ 0 \rightarrow H(I_\mathfrak {p}) \rightarrow H(V^k(\mathfrak {p})) \rightarrow H(L_k(\mathfrak {p})) \rightarrow 0 $$

is an exact sequence of $H(V^k(\mathfrak {p}))$-modules.

#### Proof

$\iota$ extends to an embedding of ${\mathfrak {g}}{\mathfrak {l}}_{1|1}$ into $\widehat{\mathfrak {g}}$ via the identification of $\mathfrak {g}$ with the horizontal subalgebra of $\widehat{\mathfrak {g}}$. Since $\mathfrak {p}$ is an object in ${{\mathcal {D}}}$, it follows that $\widehat{\mathfrak {p}}$ is an object in $\overline{{{\mathcal {D}}}}$. Since via this embedding any $\widehat{\mathfrak {g}}$-module becomes a ${\mathfrak {g}}{\mathfrak {l}}_{1|1}$-module, we see that *I* also belongs to $\overline{{{\mathcal {D}}}}$. Moreover, $I_\mathfrak {p}= I \cap V^k(\mathfrak {p})$ is a ${\mathfrak {g}}{\mathfrak {l}}_{1|1}$-module because $\mathfrak {p}$ is preserved by the ${\mathfrak {g}}{\mathfrak {l}}_{1|1}$ action and since $\overline{{{\mathcal {D}}}}$ is closed under submodules it too is an object in $\overline{{{\mathcal {D}}}}$.

We now apply Lemma 3.1. The cohomology of $I_\mathfrak {p}, V^k(\mathfrak {p})$ with respect to $\psi ^+$ is just its subspace of *E*-weight zero, since ${\mathfrak {g}}{\mathfrak {l}}_{1|1}$ acts semisimply on $V^k(\mathfrak {p})$ the same must be true for $L_k(\mathfrak {p})$. The claim follows since the *E*-weight zero subspace of $V^k(\mathfrak {p})$ is a subalgebra of $V^k(\mathfrak {p})$. $\square$

#### Corollary 3.3

With the above set-up, if $\mathfrak {p}$ is an object in ${{\mathcal {D}}}$ and if every element in the *E*-weight zero subspace of $I_\mathfrak {p}$ deforms to an element in $I_\epsilon$, then supercharacters coincide,

$$ \text {sch}[H(L_k(\mathfrak {p}))] = \text {sch}[L_k(\mathfrak {p})] = \text {sch}[L_k(\mathfrak {a}_\epsilon )]. $$

#### Proof

The first equality is a simple consequence of Lemma 3.1 applied to the subspaces of $L_k(\mathfrak {p})$ with a given conformal weight, which are objects in ${{\mathcal {D}}}$. To see the second equality, it suffices to compare the supercharacters of the ideals $I_\mathfrak {p}= I_0$ and $I_\epsilon$ for $\epsilon \ne 0$ — if the supercharacters of these ideals agree, then so too must the quotients because the character of $V^k(\mathfrak {a}_\epsilon )$ doesn’t depend on $\epsilon$.

To see that the supercharacter of $I_\epsilon$ doesn’t depend on $\epsilon$, we note that the $\epsilon \rightarrow 0$ limit of every nonzero element of $I_\epsilon$ is a nonzero element of $I_0 = I_\mathfrak {p}$, although it may be that not all elements of $I_0$ can be obtained via such a limit. Nonetheless, as only the *E*-weight zero subspace of $I_0$ contributes to its supercharacter and we assume that every element thereof deforms, we can conclude that the supercharacters must agree. $\square$

## Supercharacter formulae

In this section we affinize the deformation of $\mathfrak {osp}_{1|2n}$ into the principal subspace of ${\mathfrak {s}}{\mathfrak {l}}_{1|2n+1}$ and describe the resulting character identities, following [22]. For this section we fix positive integers $n, k > 0$.

### Deforming the principal subspace of ${\mathfrak {s}}{\mathfrak {l}}_{1|2n+1}$

Let $\mathfrak {p}$ denote the principal subalgebra of $\mathfrak {g}= {\mathfrak {s}}{\mathfrak {l}}_{1|2n+1}$. We will work with the following basis for ${\mathfrak {s}}{\mathfrak {l}}_{1|2n+1}$. We denote by $\Phi$ the set of roots of ${\mathfrak {s}}{\mathfrak {l}}_{2n+1}$ with respect to the Cartan subalgebra of diagonal matrices, $\Phi ^+$ the set of positive roots corresponding to the upper nilpotent subalgebra, and $\Delta$ the set of simple roots. The even generators will be denoted *E*, for the diagonal supermatrix with entries $(1,1,0,\dots , 0)$, $h_i$ the Cartan generators of ${\mathfrak {s}}{\mathfrak {l}}_{2n+1}$ corresponding to the simple roots $\alpha _i$, and $e_{\alpha }$, $f_\alpha$, for $\alpha \in \Phi ^+$; the odd generators will be denoted $\psi _a$, $\overline{\psi }^a$, $a = 0, \dots , 2n$. A basis for the subalgebra $\mathfrak {p}$ is given by the $e_\alpha$ for any $\alpha \in \Phi ^+$ together with $\psi _a$ for $a = 1, \dots , 2n$.

#### Remark 4.1

As $\mathfrak {p}$ does not include the element $\psi _0$ corresponding to the odd simple root of ${\mathfrak {s}}{\mathfrak {l}}_{1|2n+1}$, the root corresponding to $\psi _1$ can be thought of as simple, i.e. it cannot be written as the sum of roots contained in the subalgebra $\mathfrak {p}$.

For an element $x \in \mathfrak {g}$ we denote by $x_j = x \otimes t^j$ the corresponding element of $\widehat{\mathfrak {g}}$. We will find the following formal series particularly useful: for any $\alpha \in \Phi ^+$ we denote

$$ e_\alpha (z) = \sum _{j \in {{\textbf{Z}}}} e_{\alpha , j} z^{-j-1} \qquad f_\alpha (z) = \sum _{j \in {{\textbf{Z}}}} f_{\alpha , j} z^{-j-1} $$

and for any $a = 0, \dots 2n$ we denote

$$ \psi _a(z) = \sum _{j \in {{\textbf{Z}}}} \psi _{a, j} z^{-j-1} \qquad \overline{\psi }^a(z) = \sum _{j \in {{\textbf{Z}}}} \overline{\psi }^a_{j} z^{-j-1} $$

We also denote $e_i(z) = e_{\alpha _i}(z)$ and $f_i(z) = f_{\alpha _i}(z)$, $i = 1, \dots , 2n$.

Extending the deformation introduced in Section 2.3 to the affine setting is straightforward. We denote by $\widehat{\mathfrak {k}}_\epsilon$ the central extension of $\mathfrak {k}_{\epsilon } \otimes {{\textbf{C}}}[t^{\pm 1}]$ by ${{\textbf{C}}}K$ obtained by restricting to $X \in \mathfrak {k}_\epsilon$. The following result is an immediate consequence of Lemma 2.8.

#### Corollary 4.2

The Lie algebra $\widehat{\mathfrak {k}}_\epsilon$ tends to $\widehat{\mathfrak {p}}$ as $\epsilon \rightarrow 0$ and can be identified with $\widehat{\mathfrak {osp}}_{1|2n}$ when $\epsilon \ne 0$.

#### Remark 4.3

We note that this example fits into the general setting of Section 3. The embedding of ${\mathfrak {g}}{\mathfrak {l}}_{1|1}$ into $\mathfrak {g}= {\mathfrak {s}}{\mathfrak {l}}_{1|2n+1}$ is given the bosonic supermatrices

$$ N = \left( \begin{array}{c | c c c c} \tfrac{2n+1}{2n} &  0 &  0 &  \dots &  0\\ \hline 0 &  \tfrac{1}{2n} &  0 &  \dots &  0\\ 0 &  0 &  \tfrac{1}{2n} &  \dots &  0\\ \vdots &  \vdots &  \vdots &  \ddots &  \vdots \\ 0 &  0 &  0 &  \dots &  \tfrac{1}{2n}\\ \end{array}\right) \qquad E = \left( \begin{array}{c | c c c c} 1 &  0 &  0 &  \dots &  0\\ \hline 0 &  1 &  0 &  \dots &  0\\ 0 &  0 &  0 &  \dots &  0\\ \vdots &  \vdots &  \vdots &  \ddots &  \vdots \\ 0 &  0 &  0 &  \dots &  0\\ \end{array}\right) $$

and the fermionic supermatrices

$$ \psi ^+ = \left( \begin{array}{c | c c c c} 0 &  1 &  0 &  \dots &  0\\ \hline 0 &  0 &  0 &  \dots &  0\\ 0 &  0 &  0 &  \dots &  0\\ \vdots &  \vdots &  \vdots &  \ddots &  \vdots \\ 0 &  0 &  0 &  \dots &  0\\ \end{array}\right) \qquad \psi ^- = \left( \begin{array}{c | c c c c} 0 &  0 &  0 &  \dots &  0\\ \hline 1 &  0 &  0 &  \dots &  0\\ 0 &  0 &  0 &  \dots &  0\\ \vdots &  \vdots &  \vdots &  \ddots &  \vdots \\ 0 &  0 &  0 &  \dots &  0\\ \end{array}\right) \,. $$

It is clear that the principal subspace $\mathfrak {p}$ indeed belongs to ${{\mathcal {D}}}$. We note that this copy of ${\mathfrak {g}}{\mathfrak {l}}_{1|1}$ does not preserve $\mathfrak {k}_\epsilon$ for general $\epsilon$.

Before proving our main result, we provide a concrete description of $L_k(\mathfrak {k}_\epsilon )$ for nonzero $\epsilon$, which will follow from the following simple lemma.

#### Lemma 4.4

For $N>1$ and $k \in {{\textbf{Z}}}$, $L_k({\mathfrak {s}}{\mathfrak {l}}_N)$ is a vertex subalgebra of $L_k({\mathfrak {s}}{\mathfrak {l}}_{1|N})$.

#### Proof

The statement is proven for $k=1$ in Theorem 5.5 of [8], see also [15]. For $k>1$ one has the well-known embedding $\iota : L_k({\mathfrak {s}}{\mathfrak {l}}_N) \hookrightarrow L_1({\mathfrak {s}}{\mathfrak {l}}_{N})^{\otimes k} \hookrightarrow L_1({\mathfrak {s}}{\mathfrak {l}}_{1|N})^{\otimes k}$. For *x* in ${\mathfrak {s}}{\mathfrak {l}}_{1|N}$ let $J^x_a$ be the corresponding field in the *a*-th factor of $L_1({\mathfrak {s}}{\mathfrak {l}}_{1|N})^{\otimes k}$, so that $J^x_1 + \dots + J^x_k$ generates a homomorphic image of $V^k({\mathfrak {s}}{\mathfrak {l}}_{1|N})$. Then the image of $\iota$ is generated by the $J^x_1 + \dots + J^x_k$ for $x \in {\mathfrak {s}}{\mathfrak {l}}_N \subset {\mathfrak {s}}{\mathfrak {l}}_{1|N}$ and so $L_k({\mathfrak {s}}{\mathfrak {l}}_N)$ is in particular a vertex subalgebra of the simple quotient $L_k({\mathfrak {s}}{\mathfrak {l}}_{1|N})$. $\square$

#### Corollary 4.5

For $\epsilon \ne 0$ the quotient $L_k(\mathfrak {k}_{\epsilon })$ is isomorphic to $L_k(\mathfrak {osp}_{1|2n})$.

#### Proof

We start by noting that $L_k(\mathfrak {k}_\epsilon )$ is a homomorphic image of $V^k(\mathfrak {osp}_{1|2n})$ due to Corollary 4.2, hence contains a homomorphic image of $V^k({\mathfrak {s}}{\mathfrak {p}}_{2n})$. The above Lemma ensures that the $(k+1)$st powers of the nilpotent generators of this subalgebra vanish and it is therefore isomorphic to $L_k({\mathfrak {s}}{\mathfrak {p}}_{2n})$. The corollary is then an immediate consequence of Theorem 4.5.2 of [13], as explained in the proof of Theorem 5.3 thereof. $\square$

### The main theorem

We now establish a relatively simple variant of the main result of [22].

#### Theorem 4.6

The supercharacter of $L_k(\mathfrak {osp}_{1|2n})$ is equal to supercharacter of principal subspace of $L_k({\mathfrak {s}}{\mathfrak {l}}_{1|2n+1})$ and to the character of the principal subspace of $L_k({\mathfrak {s}}{\mathfrak {l}}_{2n})$.

#### Proof

In light of Proposition 4.5, it suffices to show that Corollary 3.3 applies to this setting. Namely, that every element of the *E*-weight zero subspace of $I_\mathfrak {p}$ can be deformed to $I_\epsilon$.

To show this, we first note that the *E*-weight zero subspace of $V^k(\mathfrak {p})$ is precisely the universal principal subspace of ${\mathfrak {s}}{\mathfrak {l}}_{2n}$. This follows from the fact that the *E*-weight zero subspace of $\mathfrak {p}$ is precisely the nilpotent subsalgebra of ${\mathfrak {s}}{\mathfrak {l}}_{2n}$. Using Lemma 4.4, it is immediate that the *E*-weight zero subspace of $I_\mathfrak {p}$ is precisely the defining ideal of the principal subspace of $L_k({\mathfrak {s}}{\mathfrak {l}}_{2n})$. The generators of this ideal are stated by Feigin-Stoyanovsky [21] for all ${\mathfrak {s}}{\mathfrak {l}}_m$ (see also [19]) and proven only for ${\mathfrak {s}}{\mathfrak {l}}_2$; the case of ${\mathfrak {s}}{\mathfrak {l}}_3$ is proven by Sadowski [18] and we show that this continues to hold for all *m* in Appendix B following work of Butorac-Kožić providing an analogous presentation for types *D*, *E*, and *F* [4]. Explicitly, the ideal defining the principal subspace of $L_k({\mathfrak {s}}{\mathfrak {l}}_{2n})$ is generated by the modes of the fields $e_{i}(z)^{k+1}$.

To complete the proof, we show that each of these generators can be deformed away from $\epsilon = 0$. We claim that this is done by simply replacing $e_i(z)^k$ by $e_{i,\epsilon }(z)^{k+1}$, where $e_{i, \epsilon }(z) = e_{i}(z) - \epsilon ^2 f_{i+1}(z)$ for $i = 2, \dots 2n-1$, $e_{2n,\epsilon }(z) = e_{\alpha _{2n}}(z)$. That these fields correspond to states in the *E*-weight zero subspace of $V^k(\mathfrak {k}_{\epsilon })$ is immediate and so it suffices to show that they belong to the *E*-weight zero subspace of *I*, i.e. the maximal ideal of $L_k({\mathfrak {s}}{\mathfrak {l}}_{2n})$, but this follows as in [22] from the fact that $e_{i,\epsilon }$ is nilpotent. $\square$

The homology of $\mathfrak {p}$ is isomorphic to the nilpotent subalgebra $\mathfrak {n}$ of ${\mathfrak {s}}{\mathfrak {l}}_{2n}$ and hence $H(V^k(\mathfrak {p}))$ is isomorphic to $V^k(\mathfrak {n})$. The last paragraph of this proof shows that the homology $H(I_\mathfrak {p})$ of the ideal $I_\mathfrak {p}$ defining the principal subspace of $L_k({\mathfrak {s}}{\mathfrak {l}}_{1|2n+1})$ is precisely the ideal defining the principal subspace of $L_k({\mathfrak {s}}{\mathfrak {l}}_{2n})$. Applying Lemma 3.2, we obtain an explicit description of the homology $H(L_k(\mathfrak {p}))$.

#### Corollary 4.7

The homology $H(L_k(\mathfrak {p}))$ of the principal subspace of $L_k({\mathfrak {s}}{\mathfrak {l}}_{1|2n+1})$ is isomorphic to the principal subspace of $L_k({\mathfrak {s}}{\mathfrak {l}}_{2n})$.

We end by noting that Conjecture 1.1 (for $p = k$) of [23] is a consequence of Theorem 4.6. Namely, we recognize the right-hand side of this conjecture as the supercharacter of $L_k(\mathfrak {osp}_{1|2n})$, see Appendix A for more details, and the left-hand side as the character of the principal subspace of $L_k({\mathfrak {s}}{\mathfrak {l}}_{2n})$, cf. [10, 21]. Equating these *q*-series leads to the desired result.

#### Corollary 4.8

(Conj. 1.1 of [23]) For $n, k \ge 0$,

4.1 $$\begin{aligned} \frac{1}{(q)_\infty ^{n(2n-1)}}\sum _{u \in {{\textbf{Z}}}^n}(-1)^{|u|} \xi (u) q^{(k+n+\scriptstyle {\frac{1}{2}})|\!|u|\!|^2+ \tilde{\rho }\cdot u} = \sum _{m \in {\mathbb {N}}^{k(2n-1)}} \frac{q^{\scriptstyle {\frac{1}{2}}\sum \limits _{i,j=1}^{k}\sum \limits _{a,b=1}^{2n-1} T_{ij} A_{ab} m_{ia} m_{jb}}}{\prod \limits _{i=1}^k \prod \limits _{a=1}^{2n-1} (q)_{m_{ia}}} \end{aligned}$$

where |*u*| denotes the sum of the components of *u*, $|\!|u|\!|^2 = u \cdot u$ the squared norm of *u*, $\tilde{\rho }$ the vector with components $\tilde{\rho }_m = m - \frac{1}{2}$, and finally

$$ \xi (u) = \prod _{1 \le l < m \le n} \frac{v_m^2 - v_l^2}{\tilde{\rho }_m^2 - \tilde{\rho }_l^2} \qquad v_m = \tilde{\rho }_m + (2(n+k)+1)u_m\,. $$

## Data Availability

The datasets generated and analyzed during the current study are available from the corresponding author on reasonable request.

## Appendix A. Some background on $\mathfrak {osp}_{1|2n}$

We follow the appendix A of [7] and use the following notation:

- $$\mathfrak {g}=\mathfrak {osp}_{1|2n}$$,
- $(\cdot |\cdot )$ a non-degenerate consistent supersymmetric invariant bilinear form,
- $\mathfrak {h}$ a Cartan subalgebra of $\mathfrak {osp}_{1|2n}$,
- $\Delta$ the root system of $\mathfrak {osp}_{1|2n}$ with respect to $\mathfrak {h}^*$,
- $\Pi =\{\alpha _1, \ldots , \alpha _n\}$ a set of simple roots of $\Delta$,
- $\Delta ^+_0$ and $\Delta ^+_1$ the sets of positive even and odd roots.

Then the highest root of $\mathfrak {osp}_{1|2n}$ is equal to $\theta = 2\alpha _1 + \cdots + 2\alpha _n$. We take $\alpha _n$ to be the (unique) non-isotropic odd simple root. The bilinear form $(\cdot |\cdot )$ on $\mathfrak {osp}_{1|2n}$ is normalized as $(\theta |\theta ) = 2$, and the $\alpha _i$ satisfy

$$\begin{aligned}&(\alpha _i|\alpha _i) = 1,\quad (\alpha _i|\alpha _{i+1}) = -\frac{1}{2},\quad i=1, \ldots , n-1,\\&(\alpha _n|\alpha _n) = \frac{1}{2},\quad (\alpha _i|\alpha _j) = 0,\quad |i-j|>1. \end{aligned}$$

The Weyl group is defined to be the Weyl group of the even subalgebra ${\mathfrak {s}}{\mathfrak {p}}_{2n}$. We identify ${\mathfrak {h}}^*$ with ${\mathfrak {h}}$ as usual, that is via $\nu : {\mathfrak {h}}^* \rightarrow {\mathfrak {h}}$ given by $(\nu (\alpha )|h) = \alpha (h)$

### A.1 The Weyl (super)dimension formula

The character $\chi _\lambda$ of a finite-dimensional irreducible module of $\mathfrak {osp}_{1|2n}$ of highest-weight $\lambda$ is given by [9, Theorem 2.35]

$$ \chi _\lambda = \frac{\prod \limits _{\alpha \in \Delta ^+_1} (1 + e^{-\alpha })}{\prod \limits _{\alpha \in \Delta ^+_0} (1 - e^{-\alpha })} \sum _{w \in W} (-1)^{\ell (w)} e^{w(\lambda +\rho ) -\rho } $$

while the supercharacter is obtained by changing the sign for every $e^\mu$ with $\mu \notin \lambda + Q_0$ for $Q_0$ the root lattice of ${\mathfrak {s}}{\mathfrak {p}}_{2n}$. A Weyl reflection satisfies $\omega (\mu ) \in \alpha _n + Q_0$ for any $\mu \in \alpha _n + Q_0$. The reflection satisfies $\omega (\rho ) - \rho \in Q_0$ if $\omega$ is a Weyl reflection corresponding to a short even root; otherwise $\omega (\rho ) - \rho$ is in $Q_0 + \alpha _n$.

Let $(-1)^{\widetilde{\ell }(w)}$ be equal to one if *w* is a product of an even number of Weyl reflections corresponding to short roots times some number of reflections corresponding to long roots. Otherwise, we set $(-1)^{\widetilde{\ell }(w)}$ to minus one. Hence, the supercharacter $\widetilde{\chi }_\lambda$ for $\lambda \in Q$ and even highest-weight vector is given by

$$ \widetilde{\chi }_\lambda = \frac{\prod \limits _{\alpha \in \Delta ^+_1} (1 - e^{-\alpha })}{\prod \limits _{\alpha \in \Delta ^+_0} (1 - e^{-\alpha })} \sum _{w \in W} (-1)^{\widetilde{\ell }(w)} e^{w(\lambda +\rho ) -\rho }. $$

#### Corollary A.1

Let $\rho _\lambda$ be an irreducible finite-dimensional highest-weight module of $\mathfrak {osp}_{1|2n}$ with highest weight $\lambda$ in the root lattice *Q* of $\mathfrak {osp}_{1|2n}$ and such that the highest-weight vector is even. Then

$$ \text {dim}(\rho _\lambda ) = \prod _{\alpha \in \Delta _0^+} \frac{(\alpha | \lambda +\rho )}{(\alpha |\rho )}, \qquad \text {sdim}(\rho _\lambda ) = \prod _{\alpha \in \Delta _{0, \text {short}}^+} \frac{(\alpha | \lambda +\rho )}{(\alpha |\rho )} $$

where $\Delta _{0, \text {short}}^+$ is the set of short even positive roots.

#### Proof

Let $A_\mu = \sum _{w \in W} (-1)^{\ell (w)} e^{w(\mu ) -\rho }$ and ${\widetilde{A}}_\mu = \sum _{w \in W} (-1)^{\widetilde{\ell }(w)} e^{w(\mu ) -\rho }$. Since $\chi _0 = 1 = \widetilde{\chi }_0$ it follows that

$$\begin{aligned}&A_\rho = \frac{\prod \limits _{\alpha \in \Delta ^+_0} (1 - e^{-\alpha })}{\prod \limits _{\alpha \in \Delta ^+_1} (1 + e^{-\alpha })} = \prod \limits _{\alpha \in \Delta ^+_0} (1 - e^{-\frac{\alpha }{(\alpha |\alpha )}}), \\&\qquad {\widetilde{A}}_\rho = \frac{\prod \limits _{\alpha \in \Delta ^+_0} (1 - e^{-\alpha })}{\prod \limits _{\alpha \in \Delta ^+_1} (1 - e^{-\alpha })}= \prod \limits _{\alpha \in \Delta ^+_0} (1 + (-1)^{(\alpha |\alpha )} e^{-\frac{\alpha }{(\alpha |\alpha )}}). \end{aligned}$$

Thus

A.1 $$\begin{aligned} \begin{aligned} A_{\lambda + \rho }(t\rho ^\vee )&= \sum _{w \in W} (-1)^{\ell (w)} e^{(w(\lambda + \rho ) -\rho | t \rho )} = \sum _{w \in W} (-1)^{\ell (w)} e^{t(w(\lambda + \rho ) -\rho | \rho )} \\&= \sum _{w \in W} (-1)^{\ell (w)} e^{t(\lambda + \rho | w(\rho )) - t(\rho + \lambda - \lambda |\rho )} = e^{t(\lambda |\rho )} A_{\rho }(t(\lambda +\rho )^\vee ) \end{aligned} \end{aligned}$$

and analogously ${\widetilde{A}}_{\lambda + \rho }(t\rho ^\vee ) = e^{t(\lambda |\rho )} {\widetilde{A}}_{\rho }(t(\lambda +\rho )^\vee )$ hence

A.2 $$\begin{aligned} \begin{aligned} \dim (\rho _\lambda )&= \lim _{t \rightarrow 0} \frac{A_{\lambda +\rho }(t\rho ^\vee )}{A_\rho (t\rho ^\vee )} = \lim _{t \rightarrow 0} \frac{A_{\rho }(t(\lambda +\rho )^\vee )}{A_\rho (t\rho ^\vee )} = \prod _{\alpha \in \Delta _0^+} \frac{(\alpha | \lambda +\rho )}{(\alpha |\rho )} \\ \text {sdim}(\rho _\lambda )&= \lim _{t \rightarrow 0} \frac{{\widetilde{A}}_{\lambda +\rho }(t\rho ^\vee )}{{\widetilde{A}}_\rho (t\rho ^\vee )} = \lim _{t \rightarrow 0} \frac{{\widetilde{A}}_{\rho }(t(\lambda +\rho )^\vee )}{{\widetilde{A}}_\rho (t\rho ^\vee )} = \prod _{\alpha \in \Delta _{0, \text {short}}^+} \frac{(\alpha | \lambda +\rho )}{(\alpha |\rho )} \end{aligned} \end{aligned}$$

$$\square $$

We note that (A.2) holds for any $\lambda \in Q \otimes _{{\textbf{Z}}}{{\textbf{C}}}$,

A.3 $$\begin{aligned} \begin{aligned} \lim _{t \rightarrow 0} \frac{A_{\lambda +\rho }(t\rho ^\vee )}{A_\rho (t\rho ^\vee )}&= \lim _{t \rightarrow 0} \frac{A_{\rho }(t(\lambda +\rho )^\vee )}{A_\rho (t\rho ^\vee )} = \prod _{\alpha \in \Delta _0^+} \frac{(\alpha | \lambda +\rho )}{(\alpha |\rho )} \\ \lim _{t \rightarrow 0} \frac{{\widetilde{A}}_{\lambda +\rho }(t\rho ^\vee )}{{\widetilde{A}}_\rho (t\rho ^\vee )}&= \lim _{t \rightarrow 0} \frac{{\widetilde{A}}_{\rho }(t(\lambda +\rho )^\vee )}{{\widetilde{A}}_\rho (t\rho ^\vee )} = \prod _{\alpha \in \Delta _{0, \text {short}}^+} \frac{(\alpha | \lambda +\rho )}{(\alpha |\rho )} \end{aligned} \end{aligned}$$

### A.2 The supercharacter of $L_k(\mathfrak {osp}_{1|2n})$ for $k \in {{\textbf{Z}}}_{>0}$

Let $\widehat{\mathfrak {g}}=\mathfrak {osp}_{1|2n}[t, t^{-1}] \oplus {{\textbf{C}}}K \oplus {{\textbf{C}}}D$ be the (untwisted) affine Lie superalgebra of $\mathfrak {osp}_{1|2n}$. The Lie superbrackets are

$$\begin{aligned}&[a\otimes t^{m_1}, b\otimes t^{m_2}] = [a,b]\otimes t^{m_1+m_2} + m_1(a|b)\delta _{m_1+m_2,0}K,\\&[D, a\otimes t^{m_1}]=m_1 a\otimes t^{m_1},\quad [K, \widetilde{\mathfrak {g}}]=0, \qquad a, b \in \mathfrak {osp}_{1|2n}, \ \ m_1, m_2 \in {{\textbf{Z}}}. \end{aligned}$$

Let $\widehat{\mathfrak {h}} = \mathfrak {h} \oplus {{\textbf{C}}}K\oplus {{\textbf{C}}}D$ be a Cartan subalgebra of $\widehat{\mathfrak {g}}$. The bilinear form on $\mathfrak {h}$ extends to $\widehat{\mathfrak {h}}$ via $(K|D)=1$ and $(K|K) = (D|D) = (h|K) = (h|D) =0$ for all $h\in \mathfrak {h}$. Let $\widehat{\Delta }$ be the root system of $\widehat{\mathfrak {g}}$ with respect to $\widehat{\mathfrak {h}}^*$ and $\widehat{\Pi } = \{\alpha _0\}\sqcup \Pi$ be a set of simple roots of $\widehat{\Delta }$. The imaginary root is $\delta :=\alpha _0+\theta$ in $\widehat{\Delta }$ and $\Lambda _0 \in \widetilde{\mathfrak {h}}^*$ such that $\delta (D)=\Lambda _0(K)=1$ and $\delta (h)=\Lambda _0(h)=\delta (K)=\Lambda _0(D)=0$ for all $h \in \mathfrak {h}$. We have $\widehat{\mathfrak {h}}^* = \mathfrak {h}^* \oplus {{\textbf{C}}}\delta \oplus {{\textbf{C}}}\Lambda _0$, $\widehat{\nu }(\delta )=K$ and $\widehat{\nu }(\Lambda _0)=D$. Denote by $\alpha ^\vee = 2\alpha /(\alpha |\alpha ) \in \widehat{\mathfrak {h}}^*$ for $\alpha \in \widehat{\mathfrak {h}}^*$ if $(\alpha |\alpha ) \ne 0$ (here we identify $\widehat{\mathfrak {h}}^*$ with $\widehat{\mathfrak {h}}$ as done in [14]). Let $\rho$ be the Weyl vector of $\mathfrak {osp}_{1|2n}$, that is $(\rho |\alpha _i^\nu )=1$ for $i=1, \dots , n$ and set $\widehat{\rho }= \rho + (n+\frac{1}{2})\Lambda _0$, where $n+\frac{1}{2}$ is the dual Coxeter number of $\mathfrak {osp}_{1|2n}$. Let $\lambda = k\Lambda _0$ for $k \in {{\textbf{Z}}}_{>0}$, then

$$ (\lambda + \widehat{\rho }) ((\alpha + m\delta )^\vee ) = (\lambda + \widehat{\rho })( \alpha ^\vee + \frac{2mK}{\alpha ^2}) = \frac{2m}{\alpha ^2]} (k+ n +\frac{1}{2}) + \rho (\alpha ^\vee ). $$

Thus

$$ \Delta ^\lambda _{1} = \{ \alpha \in \widehat{\Delta }_1 | (\lambda + \widehat{\rho })(\alpha ^\vee ) \in {{\textbf{Z}}}_{\text {odd}}\} = \{ \alpha ^\vee + m\delta | \alpha \in \Delta _1, m \in 2{{\textbf{Z}}}\} $$

and set

$$ \Delta ^\lambda _{0} = \{ \alpha \in \widehat{\Delta }_0, \frac{\alpha }{2} \notin \Delta _1 | (\lambda + \widehat{\rho })(\alpha ^\vee ) \in {{\textbf{Z}}}\} \cup \frac{1}{2}\Delta ^\lambda _0, \qquad \Delta ^\lambda = \Delta ^\lambda _{0} \cup \Delta ^\lambda _{1} $$

Then the Weyl group $W^\lambda$ is generated by all the reflections $r_{\alpha ^\vee }$ for $\alpha$ in $\Delta ^\lambda$. We observe that this group is the semi-direct product of the finite Weyl group *W* with the translations

$$\begin{aligned} t_\alpha (\lambda ):=\lambda +\lambda (K)\alpha -((\lambda |\alpha )+\frac{1}{2}(\alpha |\alpha )\lambda (K))\delta ,\qquad \text {for} \ \alpha \in Q^\vee . \end{aligned}$$

The odd positive roots are $\epsilon _n:= \alpha _n, \epsilon _{n-1}:= \alpha _{n-1} +\epsilon _n, \dots , \epsilon _1:= \alpha _1 + \epsilon _2$. They satisfy $(\epsilon _i| \epsilon _j) = \frac{1}{2}\delta _i \delta _j$, the set of short even roots is $\{ \pm \epsilon _i \pm \epsilon _j| i \ne j \}$ and the long ones are $2\epsilon _i$ for $i=1, \dots , n$; and so $Q\cong \frac{1}{\sqrt{2}} {{\textbf{Z}}}^n$. Similarly $Q^\vee \cong \sqrt{2}{{\textbf{Z}}}^n$ is generated by $2\epsilon _1, \dots , 2\epsilon _n$. The Weyl vector is

$$ \rho = \frac{1}{2}\left( \epsilon _n + 3 \epsilon _{n-1} + \dots + (2n-1)\epsilon _1 \right) $$

By [14, Theorem $1^s$] the character and supercharacter of $L_k(\mathfrak {osp}_{1|2n})$ satisfies

A.4 $$\begin{aligned} \begin{aligned} \text {ch}[L_k(\mathfrak {osp}_{1|2n})]&= \sum _{w \in W^\lambda }(-1)^{\ell (w)}\text {ch}[M(w.\lambda )] \\ \text {sch}[L_k(\mathfrak {osp}_{1|2n})]&= \sum _{w \in W^\lambda }(-1)^{\widetilde{\ell }(w)}\text {sch}[M(w.\lambda )] \end{aligned} \end{aligned}$$

with $M(\mu )$ the Verma module of highest-weight $\mu$ and $w.\lambda = w(\lambda +\rho )-\rho$ The character and supercharacter of $M(\mu )$ is

$$\begin{aligned}&\text {ch}[M(\mu )] = \frac{q^{h_\mu - \frac{c_k}{24}} D^+}{A_\rho }, \qquad \ \text {sch}[M(\mu )] = \frac{(-1)^{|v_\mu |} q^{h_\mu - \frac{c_k}{24}} D^-}{{\widetilde{A}}_\rho },\\&\qquad D^\pm := \frac{\prod \limits _{\begin{array}{c} n=1 \\ \alpha \in \Delta _1 \end{array}}^\infty (1 \pm e^{-\alpha }q^n)}{ \prod \limits _{\begin{array}{c} n=1 \\ \alpha \in \Delta _0 \end{array}}^\infty (1 - e^{-\alpha }q^n)} \end{aligned}$$

with $v_\mu$ the parity of the highest-weight vector and

$$ h_\mu = \frac{(\mu |\mu +2\rho )}{2\kappa }, \qquad c_k = \frac{kn(2n-1)}{2\kappa }, \qquad \kappa = k+n+\frac{1}{2}. $$

Thus

A.5 $$\begin{aligned} \begin{aligned} \text {ch}[L_k(\mathfrak {osp}_{1|2n})]&= \frac{D^+}{A_\rho } q^{-\frac{c_k}{24}}\sum _{w \in W, \lambda \in \kappa Q^\vee }(-1)^{\ell (w)} e^{w(\lambda +\rho ) -\rho }q^{h_\lambda } \\&= D^+ q^{-\frac{c_k}{24}}\sum _{\lambda \in \kappa Q^\vee } \frac{A_{\lambda +\rho }}{A_\rho } q^{h_\lambda } \\ \text {sch}[L_k(\mathfrak {osp}_{1|2n})]&= \frac{D^-}{{\widetilde{A}}_\rho } q^{-\frac{c_k}{24}}\sum _{w \in W, \lambda \in \kappa Q^\vee }(-1)^{\widetilde{\ell }(w) + 2\lambda ^2} e^{w(\lambda +\rho ) -\rho }q^{h_\lambda } \\&= D^- q^{-\frac{c_k}{24}}\sum _{\lambda \in \kappa Q^\vee } (-1)^{2\lambda ^2}\frac{{\widetilde{A}}_{\lambda +\rho }}{{\widetilde{A}}_\rho } q^{h_\lambda } \end{aligned} \end{aligned}$$

The claimed character formula follows using (A.3), that is the left-hand side of (4.1) is the specialization of $\text {sch}[L_k(\mathfrak {osp}_{1|2n})]$,

A.6 $$\begin{aligned} \lim _{t\rightarrow 0} \text {sch}[L_k(\mathfrak {osp}_{1|2n})](t\rho ^\vee ) = \frac{1}{(q)_\infty ^{n(2n-1)}}\sum _{u \in {{\textbf{Z}}}^n}(-1)^{|u|} \xi (u) q^{(k+n+\scriptstyle {\frac{1}{2}})|\!|u|\!|^2+ \tilde{\rho }\cdot u} \end{aligned}$$

where |*u*| denotes the sum of the components of *u*, $|\!|u|\!|^2 = u \cdot u$ the squared norm of *u*, $\tilde{\rho }$ the vector with components $\tilde{\rho }_m = m - \frac{1}{2}$, and finally

$$ \xi (u) = \prod _{1 \le l < m \le n} \frac{v_m^2 - v_l^2}{\tilde{\rho }_m^2 - \tilde{\rho }_l^2} \qquad v_m = \tilde{\rho }_m + (2(n+k)+1)u_m\,. $$

## Appendix B. Presentation for the principal subspace of $L_k({\mathfrak {s}}{\mathfrak {l}}_{N+1})$

In this appendix we provide a presentation of the principal subspace of $L_k({\mathfrak {s}}{\mathfrak {l}}_{N+1})$, following the work of Georgiev [10] and of Butorac-Kožić [4].

We start with some notation, which largely overlaps with that of the main text. Let $\mathfrak {g}= {\mathfrak {s}}{\mathfrak {l}}_{N+1}$ and denote by $\widehat{\mathfrak {g}}$ its affinization. For $X \in \mathfrak {g}$ denote by $X_m = X \otimes t^m \in \widehat{\mathfrak {g}}$. Choose a triangular decomposition $\mathfrak {g}= \mathfrak {n}_+ \oplus \mathfrak {h}\oplus \mathfrak {n}_-$; we set $\bar{\mathfrak {n}}_\pm = \mathfrak {n}_\pm \otimes {{\textbf{C}}}[t]$. Let $\Delta$ denote the set of roots with respect to $\mathfrak {h}$, $\Delta _\pm$ the set of positive/negative roots, and $\Pi = \{\alpha _1, \dots , \alpha _N\}$ the set of positive simple roots. We will make use of the Chevalley basis $\{x_\alpha \}_{\alpha \in \Delta } \cup \{h_{\alpha _i}\}_{i=1}^N$.

We denote by $v_k$ the highest weight vector of the simple vacuum module $L_k({\mathfrak {s}}{\mathfrak {l}}_{N+1})$ at level *k*. As defined by Feigin-Stoyanovsky [21], the principal subspace of $L_k({\mathfrak {s}}{\mathfrak {l}}_{N+1})$ is

$$ W_{L_k({\mathfrak {s}}{\mathfrak {l}}_{N+1})} := U(\bar{\mathfrak {n}}_+)v_k $$

where $U(\mathfrak {f})$ denotes the universal enveloping algebra of a Lie algebra $\mathfrak {f}$. The principal subspace is clearly a quotient of $U(\bar{\mathfrak {n}}_+)$; the aim of this appendix is to show that the left ideal ${\mathcal {I}}_k$ annihilating $v_k$ is given by

$$ {\mathcal {I}}_k = U(\bar{\mathfrak {n}}_+) \bar{\mathfrak {n}}^{\ge 0}_+ + \sum _{i=1}^N \sum _{m \ge k+1} U(\bar{\mathfrak {n}}_+) R_i(-m)\,, $$

where $$\bar{\mathfrak {n}}^{\ge 0}_+ = \mathfrak {n}\otimes {{\textbf{C}}}[t]$$ and

$$ R_i(-m) = \sum _{\begin{array}{c} m_1, \dots , m_{k+1} \le -1\\ m_1 + \dots + m_{k+1} = -m \end{array}} x_{\alpha _i, m_1} \dots x_{\alpha _i, m_{k+1}}\,. $$

This gives us the desired presentation:

### Theorem B.1

For all positive integer integers we have

$$ W_{L_k({\mathfrak {s}}{\mathfrak {l}}_{N+1})} \simeq U(\bar{\mathfrak {n}}_+) / {\mathcal {I}}_k\,. $$

### B.1 Quasiparticle basis of $W_{L_k({\mathfrak {s}}{\mathfrak {l}}_{N+1})}$

We now introduce a basis of $W_{L_k({\mathfrak {s}}{\mathfrak {l}}_{N+1})}$ due to [10]. We first consider the formal series

$$ x_{\alpha _i}(z) = \sum _{m} x_{\alpha _i, m} z^{-m-1}\,, \qquad i = 1, \dots , N\,, $$

where are fields on $L_k({\mathfrak {s}}{\mathfrak {l}}_{N+1})$. Note that $[x_{\alpha _i}(z_1), x_{\alpha _i}(z_2)] = 0$, so that

$$ x_{n \alpha _i}(z) = x_{\alpha _i}(z)^n = \sum _{m} x_{n \alpha _i, m} z^{-m-n} $$

is also a well-defined field on $L_k({\mathfrak {s}}{\mathfrak {l}}_{N+1})$. Note that the $R_i(-m)$ appearing in the definition of ${\mathcal {I}}_k$ are coefficients of $x_{(k+1) \alpha _i}(z)$. We also note that by the Poincaré-Birkoff-Witt theorem for $U(\bar{\mathfrak {n}}_+)$ we have the following isomorphism of vector spaces:

$$ U(\bar{\mathfrak {n}}_+) = U(\bar{\mathfrak {n}}_{\alpha _N}) \dots U(\bar{\mathfrak {n}}_{\alpha _1}) $$

where $\bar{\mathfrak {n}}_{\alpha _i} = \mathfrak {n}_{\alpha _i} \otimes {{\textbf{C}}}[t^{\pm 1}]$ and $\mathfrak {n}_{\alpha _i} = {{\textbf{C}}}x_{\alpha _i}$ is the 1-dimensional abelian Lie algebra generated by the root vector $x_{\alpha _i}$.

As in [10], we call the coefficient $x_{n \alpha _i, m}$ a quasiparticle of color *i*, charge *n*, and energy $-m$. We also call an endomorphism of the form

$$ b = \big (x_{n_{r_N,N}\alpha _N, m_{r_N, N}} \dots x_{n_{1,N}\alpha _N, m_{1, N}}\big ) \dots \big (x_{n_{r_1,1}\alpha _1, m_{r_1, 1}} \dots x_{n_{1,1}\alpha _1, m_{1, 1}}\big ) $$

a quasiparticle monomial; we say that such a quasiparticle monomial has color-charge-type

$$ (n_{r_N,N}, \dots , n_{1,N}; \dots ; n_{r_1,1}, \dots , n_{1,1}) $$

and index sequence

$$ (m_{r_N,N}, \dots , m_{1,N}; \dots ; m_{r_1,1}, \dots , m_{1,1})\,. $$

We call the tuples $(n_N; \dots ; n_1)$ and $(m_N; \dots ; m_1)$, where $n_i = n_{r_i, i} + \dots + n_{1,i}$ and $m_i = m_{r_i, i} + \dots + m_{1,i}$, its color type and its index sum. As quasiparticles of color *i* commute amongst themselves, we assume that $n_{a+1, i} \le n_{a,i}$ and, if $n_{a+1, i} = n_{a,i}$, that $m_{a+1, i} \le m_{a,i}$ without loss of generality.

We will also need two orders on the set of all quasiparticle monomials: the lexicographical (linear) order < and the multidimensional (partial) order $\prec$. For two such monomials $b, b'$ we say $b < b'$ if the color-charge-type of *b* is less than that of $b'$ in lexicographical order; if their color-charge-types are the same, we say $b < b'$ if its index sequence is less than that of $b'$ in lexicographical order. Additionally, we say that $b \prec b'$ if $b < b'$ and, for every $1 \le s \le N$, one has $m_s + \dots + m_1 \le m_s' + \dots + m_1'$ and this is a strict inequality for at least one *s*, where $m_i = \sum _a m_{a,i}$.

Denote by *B* the set of quasiparticle monomials satisfying

B.1 $$\begin{aligned} m_{a+1, i}&\le m_{a, i} - 2 n_{a,i} \quad \text {if } n_{a+1, i} = n_{a,i} \end{aligned}$$

B.2 $$\begin{aligned} m_{a, i}&\le (1-2a) n_{a,i} + \sum _{b=1}^{r_{i-1}} \min (n_{b,i-1}, n_{a,i}) \end{aligned}$$

B.3 $$\begin{aligned} n_{a,i}&\le k \end{aligned}$$

for all $i = 1, \dots N$ and $a = 1, \dots r_i$.

#### Theorem B.2

( [10], Theorem 5.2) The set

$$ \mathfrak {B} = \{b v_k | b \in B\} $$

is a basis of the principal subspace $W_{L_k({\mathfrak {s}}{\mathfrak {l}}_{N+1})}$.

### B.2 Spanning set for $U(\bar{\mathfrak {n}}_+)/{\mathcal {I}}_k$

We now give a spanning set for the quotient $U(\bar{\mathfrak {n}}_+)/{\mathcal {I}}_k$, from which we will conclude Theorem B.1. For $X \in U(\bar{\mathfrak {n}}_+)$ we denote by $\overline{X}$ its image in this quotient; we then set

$$ \overline{\mathfrak {B}} = \{\overline{b} | b \in B\}\,. $$

#### Proposition B.3

The set $\overline{\mathfrak {B}}$ spans the quotient $U(\bar{\mathfrak {n}}_+)/{\mathcal {I}}_k$.

We will prove this proposition briefly, first using it to prove the main result of this appendix as in the proof of Theorem 4.1 of [4].

#### Proof of Theorem B.1

We start with the canonical surjection

$$ f: U(\bar{\mathfrak {n}}_+) \rightarrow W_{L_k({\mathfrak {s}}{\mathfrak {l}}_{N+1})}\,, \quad X \mapsto X v_k\,. $$

It is clear that ${\mathcal {I}}_k$ belongs to the kernel of *f*, so this map factors through $U(\bar{\mathfrak {n}}_+)/{\mathcal {I}}_k$. Denote by $\overline{f}$ the corresponding map from $U(\bar{\mathfrak {n}}_+)/{\mathcal {I}}_k$ to $W_{L_k({\mathfrak {s}}{\mathfrak {l}}_{N+1})}$; this map is necessarily surjective and bijectively maps the spanning set $\overline{\mathfrak {B}}$ to the basis $\mathfrak {B}$. We conclude that $\overline{\mathfrak {B}}$ is a basis of $U(\bar{\mathfrak {n}}_+)/{\mathcal {I}}_k$ and that $\overline{f}$ is an isomorphism of vector spaces. $\square$

We end this appendix by sketching a proof of Proposition B.3. The proof closely mirrors the proof of Theorem 5.1 of [10] and parts of the proof of Theorem 4.1 of [4], so we only provide the main ideas.

#### Proof of Proposition B.3

We note three properties satisfied by the formal series $x_{n \alpha _i}(z)$ independent of the module of $\widehat{\mathfrak {g}}$ they are acting on and hence can be applied to $U(\bar{\mathfrak {n}}_+)$. The first two properties ultimately stem from the expression $x_{n \alpha _i}(z) = x_{\alpha _i}(z)^n$. The first property takes the form

B.4 $$\begin{aligned} \begin{aligned} x_{n \alpha _i}(z) x_{n' \alpha _i}(z)&= x_{(n-1) \alpha _i}(z) x_{(n'+1) \alpha _i}(z)\\&= \dots = x_{\alpha _i}(z) x_{(n+n'-1)\alpha _i}(z) = x_{(n+n')\alpha _i}(z) \end{aligned} \end{aligned}$$

for $0 < n \le n'$, cf. Eq. (3.18) of [10]; this induces *n* relations on quasiparticle monomials of color *i*. The second property we will need is given by

B.5 $$\begin{aligned} \begin{aligned} \frac{n + n'}{n}\bigg (\frac{d }{d z}x_{n \alpha _i}(z)\bigg ) x_{n' \alpha _i}(z)&= \frac{n + n'}{n-1}\bigg (\frac{d }{d z}x_{(n-1) \alpha _i}(z)\bigg ) x_{(n'+1) \alpha _i}(z)\\&= \dots = \frac{n + n'}{1}\bigg (\frac{d }{d z}x_{\alpha _i}(z)\bigg ) x_{(n+n'-1) \alpha _i}(z)\\&= \frac{d }{d z}\bigg (x_{n \alpha _i}(z) x_{n' \alpha _i}(z)\bigg ) \end{aligned} \end{aligned}$$

for $0 < n \le n'$, cf. Eq. (3.22) of [10]; this induces another *n* relations on quasiparticle monomials of color *i*.

The third and final property we will need is as follows. We consider the generating function of quasiparticle monomials of a fixed color-charge-type

$$ X_{\{n_{a,i}\}}(\{z_{a,i}\}) = x_{n_{r_N,N}}(z_{r_N,N}) \dots x_{n_{1,N}}(z_{1,N}) \dots x_{n_{r_1,1}}(z_{r_1,1}) \dots x_{n_{1,1}}(z_{1,1}) $$

and multiply it by the Laurent polynomial

$$ P_{\{n_{a,i}\}}(\{z_{a,i}\}) = \prod _{i=2}^N \prod _{a=1}^{r_i} \prod _{b=1}^{r_{i-1}} \bigg (1-\frac{z_{b,i-1}}{z_{a,i}}\bigg )^{\min (n_{b,i-1},n_{a,i})}\,. $$

The third property then says that their product, modulo ${\mathcal {I}}_k$, belongs to $(U(\bar{\mathfrak {n}}_+)/{\mathcal {I}}_k)[[\{z_{a,i}\}]]$:

B.6 $$\begin{aligned} P_{\{n_{a,i}\}}(\{z_{a,i}\}) X_{\{n_{a,i}\}}(\{z_{a,i}\}) + {\mathcal {I}}_k \in \big (U(\bar{\mathfrak {n}}_+)/{\mathcal {I}}_k\big )[[\{z_{a,i}\}]] \end{aligned}$$

cf. Lemma 5.1 of [10].

As in the proof of Theorem 5.1 of [10], the properties in Eq. (B.4) imply that if the color-charge-type of the quasiparticle monomial *b* has $n_{a,i} = n_{a+1,i}$ for some *i*, *a* but its index sequence does not satisfy

$$ m_{a+1,i} \le m_{a, i} - 2 n_{a,i} $$

then it can be re-expressed as a linear combination of quasiparticle monomials $b'$ with $b \prec b'$ of same color type and index sum, but possibly different color-charge-type; only a finite number of the $b'$ do not belong to $U(\bar{\mathfrak {n}}_+)\bar{\mathfrak {n}}_+^{\ge 0} \subset {\mathcal {I}}_k$. Thus, the first condition Eq. (B.1) defining the set *B* of quasiparticle monomials follows from the property in Eq. (B.4).

If the quasiparticle monomial *b* does not satisfy the second condition Eq. (B.2) defining *B*

$$\begin{aligned} m_{a,i} \le (1-2a) n_{a,i} + \sum _{b=1}^{r_{i-1}} \min (n_{b,i-1}, n_{a,i}) \end{aligned}$$

then the properties in Eqs. (B.4), (B.5), (B.6) imply that it can be re-expressed as a linear combination of quasiparticle monomials $b' \succ b$ of the same color type and total index sum, only a finite number of which do not belong to $U(\bar{\mathfrak {n}}_+)\bar{\mathfrak {n}}_+^{\ge 0} \subset {\mathcal {I}}_k$. This is proven exactly as in Theorem 5.1 of [10] and follows by induction on the color type and index sum.

For the last condition Eq. (B.3) defining the set *B*, we proceed as in the last paragraph in Section 5.5 of [4]. If the color-charge-type of quasiparticle monomial *b* satisfies the first two conditions Eqs. (B.1), (B.2) and has $n_{a,i} > k$ for some *a*, *i*, i.e. it does not satisfy Eq. (B.3), then the commutation relations of the $x_{n \alpha _j}$ imply that we can bring the term $x_{n_{a,i}}(z)$ in the product $P_{\{n_{a,i}\}}(\{z_{a,i}\}) X_{\{n_{a,i}\}}(\{z_{a,i}\})$ all the way to the right and hence we find that this product belongs to ${\mathcal {I}}_k$. As *b* is the coefficient of $z_{r_N, N}^{m_{r_N,N}+n_{r_N, N}}... z_{1, 1}^{m_{1,1}+n_{1, 1}}$ of $X_{\{n_{a,i}\}}(\{z_{a,i}\})$, we can take this same coefficient of $P_{\{n_{a,i}\}}(\{z_{a,i}\}) X_{\{n_{a,i}\}}(\{z_{a,i}\})$ to see that *b* can be expressed as a linear combination of quasiparticle monomials $b'$ of the same color-charge-type. Only a finite number of such $b'$ do not belong to ${\mathcal {I}}_k$, so we can continue this process to ultimately show that *b* itself belongs to ${\mathcal {I}}_k$. $\square$

## Footnotes

1 We note that the parameter *k* in [23] differs from our *k* by 1: $k_{WZ} = k + 1$.
